# Gynæcology in the Seventeenth Century

**Published:** 1880-02

**Authors:** James I. Tucker

**Affiliations:** Chicago


### ARTICLE VI.

GYNÆCOLOGY IN THE SEVENTEENTH CENTURY.* By DR. JAMES I. TUCKER, Chicago.

The opinion is too generally held that gynæcology is a branch of medicine that lay fallow and unused in the middle ages and even in more recent times, but that this is not the case there are many indications to show, among them a treatise on the diseases of women which appeared in the seventeenth century.

The book is entitled: "*L'Hydre Féminine Combatve par la Nymphe pougoise ov Traité des Maladies des femmes, Gveries par les Eaux de Pougues, par M. Avgvstin Covrrade, Docteur en médecine. A Nevers par Iean Millot, imprimeur et libraire de Son Altesse Serenissime de Mantone. MDCXXXIV.*

The author of this work is Augustin Courrade, physician to the Princess Maria von Gouzaga, afterwards Queen of Poland, who resided sometimes in Paris, sometimes in Pougues, and at the latter place made use of the medicinal springs. This work which was printed in 1634 is really an advocate of the curative properties of the waters of Pougues, but it consists of two parts, the first of which treats of the diseases of women. According to the bombastic literary taste of the period, the book not only bears the allegorical title "*L'Hydre Feminine*," but the subject is also allegorically treated. The first part contains "The seven heads of Hydra," meaning seven chapters on diseases of women.

The first head of Hydra is *Sterility.*

Sterilty does not happen to women alone, as is generally supposed. We observe it in males also, where it is called impotence, although there are sterile men who are not impotent. Sometimes the fault is neither in the man nor the woman, but in a dis-harmony between the two, the cause of which is unknown.

The function of the ovaries is of course unknown to the author. Generation was to him the result of the mixing of the male and female semen.

---

* *Annales de Gynécologie,* (1879.) *Deutsche Archiv für Geschichte der Medicine, etc.* (*Zweiten Bandes Drittes Heft.*)

The causes of sterility in women are internal and external.

To the former belong the *atmosphere* for many winds make women sterile.—Hippocrates.

*Drinking and eating in excess*, more especially drunkenness, produce sterility. The same is true of violent passions, particularly hate and jealousy.

*Immoderate bodily exercise* belongs here. The author would doubtless have spoken of dancing, but out of respect for the princess, who was very fond of this amusement, he was restrained.

*Astrological influences* are named, but he merely mentions them as they appeared to him little worthy of belief. The same is true of *magic* and *witchcraft.*

*The internal causes are seven in number.* General and particular irregularities of pregnancy. Among the first, is that which was later called *temperament.*

*Faulty condition* of the *female semen* in quality and quantity.

*Disturbances of menstruation*, which are spoken of more fully in the second head of Hydra.

The *fluor albus* (in third head of hydra.)

*Malformation of the uterus.* He mentions here occlusion of the vulva by a membrane firmer than the hymen, and a deviation of the collum uteri, which hinders the entrance of the sperma "*tortu et oblique.*"

*Diseases of the uterus.* Tumors, abscesses, callosities, etc, which are described in full in the other heads.

Sterility can be treated exclusively by "*des fumées*, *vapeurs*, *racines*, *fleures*, *herbes*, *poissons*, *animaux*," but he avoids giving these in detail lest they be misused.

The four "*Intemperies générales*" are to warmth, cold, dryness and moisture. These not seldom combine as in the atmosphere, as dry cold, warmth and moisture, dampness and cold, and dry heat. Three of these he combats with the waters of Pougues. The efficacy of these waters depends upon the nitrum which they contain, and he compares them in this respect to the waters of the Nile. The Egyptian women are prolific because they drink large quantities of the waters of the Nile which are rich in saltpeter.

The *second head of Hydra* treats of faulty menstruation, and is really only a physiology of menstruation.

The menstrual blood does not seem to him to serve as nourishment for the fœtus, as Hippocrates thought. He looks upon it as impure blood which Nature discharges.

At the time of puberty the female semen forms under the influence of a motion, which the natural warmth increases; this motion extends into the blood-vessels, through which the impure blood escapes. There is, therefore, a reciprocal relation between menstruation and the formation of female semen. Without this the woman cannot conceive. The female semen is produced during the menopause, when the blood ceases to flow.

The unfruitfulness of advanced life is explained by the loss of the functional action of the semen, through the departure of the "*esprit génératif.*" Through this circumstance the uterus becomes dry, the little mouth-like endings of its veins are obliterated, so that neither the menstrual blood nor that which nourishes the fœtus can find an outlet. The impure menstrual blood retained in the body, mixes with that which serves in the formation of semen and the nourishment of the fœtus, and hence conception is impossible.

If the menstrual flow is excessive, much good blood is lost with the impure, whereby the organism is weakened and the seed loses its "*esprit génératif.*" Thus sterility may be the consequence of menstrual disturbance.

Menstruation is suppressed by causes external and internal.

To the first belong abstemiousness in eating, drinking and in everything of a sensual nature, as we observe among religious people; passionate excitement of the mind—as fear, melancholy, love-sickness, etc.

To the internal causes belong obstructions of the vessels, which result either from failure of conformation or an immoderate collection of fat. This accumulated fat stops up the vessels, or chokes them by pressure. Ruptures and flexions of the uterus have like effect.

Profuse menstruation has various exciting causes. Either the body contains too much blood, and the vessels burst from the increased pressure, or the blood is in bad condition.

*The third head of Hydra is fluor albus.*

Under the head of fluor albus we are to understand the different fluids of the body. These fluids are the blood, the gall, the melancholy and the phlegm, and whenever any one of these is foul, the fluor albus has a different color. It is white from blood, yellow from the gall, black from melancholy, or livid from phlegm. The black flow is most seldom. The uterus has nothing to do with the flow.

From this fluid "gonorrhœa" can be contracted, which, however, must not be confounded with that which "*est contractée pour avoir monté sur les chevaux de Naples.*" This gonorrhœa is a spermatorrhœa of women, and is very dangerous.

*The fourth head of Hydra is love, nymphomania and melancholy.*

The fluids are retained in the vessels of the uterus, and this forms winds which pass up to the brain; "Hence these tears."

*The fifth head of Hydra is suffocation (erstickung) of the uterus.*

Under this head the author includes hysteria and every form of it which is accompanied by convulsions. It is caused by pressure on the diaphragm from the side of the uterus, either on account of congestion of this organ or contamination of the menstrual or seminal fluid.

*The sixth head of Hydra is formed by the pale complexion, chlorosis.*

Chlorosis is the result of retained menstrual fluid, and alteration of the taste; there is a false desire for things which do not nourish. Thus the blood is spoiled, it loses its normal color, and the tissues of the body bleach.

*The seventh head of Hydra is represented by tumors of the uterus.*

The physicians of the seventeenth century had a tolerably correct idea of the diseases of the uterus. They recognized engorgement, which they called œdema; they recognized phlegmon, carcinoma and ulcerated cancer, and congestion of the uterus, which they called erysipelas. Granulations and ulcerations of the collum uteri are described so exactly that one would think that Dr. Courrade must have known and used the speculum.

